# Parental Perspectives on Human Papillomavirus (HPV) Vaccination in Gulf Cooperation Council Countries: A systematic review

**DOI:** 10.1097/MD.0000000000040124

**Published:** 2024-10-18

**Authors:** Najim Z. Alshahrani, Jaber Abdullah Alshahrani, Badur Saad Almushari, Fahad Marzooq Alshammri, Wael Saeed Alshahrani, Ahmed Ayed Hadi Alzabali, Abdulrahman Ahmed Alshehri, Nasser Z. Alduaydi, Manea Alqarni, Ammar Mohammed A. Alamri, Khalid Alotaibi

**Affiliations:** a Department of Family and Community Medicine, Faculty of Medicine, University of Jeddah, Jeddah, Saudi Arabia; b Family Medicine and Medical Education, Armed Forces Hospital Southern Region, Khamis Mushit, Saudi Arabia; c Department of Family Medicine, General Directorate of Health Affairs in Aseer Region, Ministry of Health, Abha, Saudi Arabia; d Rafha General Hospital, Ministry of Health, Rafha, Saudi Arabia; e Armed Forces Hospital Southern Region, Khamis Mushit, Saudi Arabia; f First Riyadh Health Cluster, Ministry of Health, Riyadh, Saudi Arabia; g Internal Medicine & Hematology, Armed Forces Hospital Southern Region, Khamis Mushit, Saudi Arabia; h College of Medicine, King Khalid University, Abha, Saudi Arabia; i Prince Sultan Military College of Health Sciences, Dhahran, Saudi Arabia.

**Keywords:** GCC, Gulf Cooperation Council, HPV, parents’ attitude, parents’ knowledge, vaccine

## Abstract

**Background:**

This systematic review aims to synthesize existing research on parental knowledge, attitudes, and barriers to human papillomavirus (HPV) vaccination in the Gulf Cooperation Council (GCC) countries.

**Methods:**

Following PRISMA guidelines, this systematic review analyzed parental knowledge, attitudes, and barriers to HPV vaccination in 6 GCC countries. A comprehensive search across multiple electronic databases (Embase, Cumulative Index of Nursing and Allied Health Literature, Scopus, Ovid MEDLINE, Web of Science, and PubMed) was conducted, focusing on studies published between January 2010 and December 2023. Inclusion criteria targeted studies in English or Arabic involving parents in GCC countries, excluding non-research publications and those not using survey techniques.

**Results:**

The review included 7 studies from Saudi Arabia, the United Arab Emirates, and Qatar. No study was found in Oman, Bahrain, and Kuwait. The findings highlighted low knowledge and awareness of HPV and the vaccine, with significant variation across studies. According to our review findings, the level of awareness in the 7 studies was found to be low to high. These studies collectively illustrate a range of awareness levels, from as low as 11% awareness of the HPV-cervical cancer link to as high as 68% general awareness of HPV. Attitudes towards vaccination were poor in Saudi Arabia but more positive in the United Arab Emirates and Qatar. Major barriers included safety concerns, lack of information, and cultural beliefs.

**Conclusion:**

Despite moderate awareness in some GCC countries, substantial knowledge gaps and vaccine hesitancy persist. Targeted educational campaigns, effective communication strategies, and involvement of community leaders are essential to improve HPV vaccination uptake.

## 1. Introduction

Cervical cancer is the 4th most common cancer among women globally, with the incidence of human papillomavirus (HPV) infection, a key etiological factor, estimated to affect between 85% and 95% of women with cervical cancer worldwide.^[1–3]^ In the Gulf Cooperation Council (GCC) Countries, which include Bahrain, Kuwait, Oman, Qatar, Saudi Arabia, and the United Arab Emirates (UAE), research shows significant variations in HPV positivity rates. Qatar has the highest rate at 31.3%, followed by Bahrain at 20%, Saudi Arabia at 17.2%, and the UAE at 14.7%.^[4]^ In Saudi Arabia, cervical cancer is notably prevalent, ranking as the 11th most common cancer overall and the 9th most common among women aged 15 to 44, according to the Saudi Cancer Registry.^[5]^ HPV, a widespread sexually transmitted infection, is the primary cause of cervical cancer, with HPV types 16 and 18 responsible for approximately 70% of cervical cancers and pre-cancerous cervical lesions.^[2,3,5]^ Beyond cervical cancer, HPV is also implicated in cancers of the oropharynx, vulva, anus, vagina, and penis.^[3,5]^

The HPV vaccine, introduced by the Centers for Disease Control and Prevention and the Food and Drug Administration in 2006, has demonstrated efficacy in reducing the incidence of cervical cancer by targeting high-risk HPV types 16 and 18.^[6,7]^ In Saudi Arabia, the vaccine has been available since 2010, accessible at major healthcare facilities either free of charge or at a cost to women with a valid medical prescription.^[8]^ The vaccine is typically administered in a series of 3 doses over 6 months to females aged 11 to 26, showing significant effectiveness in preventing cervical cancer and reducing the prevalence of various HPV-related diseases.^[8,9]^ However, vaccine availability varies across the GCC; for example, Oman has not yet included the HPV vaccine in its Expanded National Immunization Program, limiting its availability to a few private institutions.^[10]^

Parents’ decisions to vaccinate their children against HPV are influenced by their perspectives on the vaccine and their socioeconomic status.^[11]^ Research highlights that socio-demographic factors, such as parents’ religion, education level, age, gender, job status, and number of children, significantly impact HPV vaccination uptake.^[11]^ Additionally, socioeconomic factors and cultural norms, including lower income and poverty, are associated with reduced HPV vaccine uptake rates.^[11,12]^ Concerns about vaccine side effects and long-term safety also affect attitudes, understanding, and health behaviors.^[7]^ In the GCC region, shared religious and cultural values play a crucial role in healthcare decisions, particularly regarding vaccinations.^[10–12]^ Investigating parental characteristics and attitudes towards the HPV vaccine in the GCC can support the implementation of the vaccine in these communities.

To date, while there has been some research on HPV vaccination within individual GCC countries, a comprehensive review synthesizing parental knowledge, attitudes, and barriers across the region is lacking. This systematic review aims to address this gap by collecting, analyzing, and integrating data from multiple studies to provide a detailed understanding of the factors influencing HPV vaccination decisions among parents in the GCC. The secondary objective is also to summarize the knowledge level of HPV across studies. This knowledge is vital for developing targeted strategies to improve vaccine uptake and address public health challenges related to HPV in these communities.

## 2. Methods and materials

The systematic review adhered to the PRISMA (Preferred Reporting Items for Systematic Reviews and Meta-Analyses) guidelines. The review focused exclusively on quantitative studies to ensure the inclusion of data that could be consistently analyzed. At every stage, including the search process, data extraction, and quality appraisal, consistency checks were performed on all studies. Any discrepancies were resolved through discussion. To manage data duplication, reference management software (EndNote X9) was used to eliminate duplicates, followed by a manual verification process.

### 2.1. Search strategy

This study aimed to investigate parental attitudes, knowledge, and barriers concerning the HPV vaccine across 6 GCC countries: Saudi Arabia, Kuwait, Bahrain, Qatar, Oman, and the UAE. A thorough search was conducted across several electronic databases, including Embase, Cumulative Index of Nursing and Allied Health Literature, Scopus, Web of Science, Ovid MEDLINE, and PubMed, to identify relevant research studies. A preliminary manual search of academic literature was conducted to identify appropriate keywords and Medical Subject Heading terms, which were then used to formulate the search strategy.

Key search terms included geographic identifiers such as “Saudi Arabia,” “Kuwait,” “Bahrain,” “Qatar,” “Oman,” and “United Arab Emirates,” combined with terms related to the HPV vaccine such as “HPV,” “papillomavirus vaccine,” and “wart virus vaccine.” To identify studies focused on parental perspectives, terms like “parent,” “parent–child relations,” and “knowledge” were used. Boolean operators (e.g., AND, OR) were applied to combine these terms effectively across all databases. The search terms were carefully selected to ensure the inclusion of studies relevant to the GCC context and are detailed in Table 1.

**Table 1:** Population, intervention, comparison, outcome (PICO) tool.

PICO	Description	Search terms and connectors
Population	Parents in GCC countries: Saudi Arabia, Kuwait, Bahrain, Qatar, Oman, and the United Arab Emirates	(“Saudi Arabia” OR “Kuwait” OR “Bahrain” OR “Qatar” OR “Oman” OR “United Arab Emirates”). tw.
Intervention	HPV vaccination	(“HPV” OR “papillomavirus vaccine” OR “wart virus vaccine”). tw.
Comparison	No comparator was used as only cross-sectional studies were included	–
Outcome	Parental knowledge, and knowledge toward HPV vaccination Secondary outcome: Summarize knowledge of HPV across studies	(“parent” OR “parent-child relations” OR “parent or child-parent relation”).tw. AND (“attitude” OR “knowledge”). tw.

### 2.2. Inclusion and exclusion criteria

The study’s inclusion criteria were specific to ensure relevance and quality. Firstly, it included studies conducted exclusively in GCC countries: Saudi Arabia, Kuwait, Bahrain, Qatar, Oman, and the UAE. Additionally, only studies published within the last decade (January 2010–December 2023) were considered to ensure the most current data. Language restrictions were applied, including only studies written in English or Arabic, reflecting the primary languages of scientific publications in these regions. The focus was on parental knowledge, and attitudes towards HPV and the HPV vaccine, requiring that these studies provide cross-sectional data to ensure the researchers’ interventions did not influence these factors. The review included only quantitative studies that used survey methodologies to collect data.

The exclusion criteria were designed to filter out irrelevant or noncontributory studies. Studies were excluded if the target population did not consist of parents, as the focus was specifically on parental perspectives. Any study whose results did not address the knowledge, attitudes, and/or behaviors of parents was also excluded. Moreover, studies that did not involve a survey technique were removed to maintain consistency in the data collection method. Qualitative studies, literature reviews, and studies using methodologies other than surveys were excluded to maintain consistency in the data collection approach.

### 2.3. Study characteristics

The studies included in this review primarily involved parents who were Arab nationals or residents of the GCC countries. There were no specific eligibility criteria regarding the marital status or age of the parents, although the age range of participants typically varied from 18 to 60 years. Surveys were conducted either online or in person, with varying response rates reported across the studies.

### 2.4. Quality assessment

Each study’s methodology was rigorously evaluated using the Appraisal Tool for Cross-Sectional Studies. This tool assesses several aspects of study quality, including design, sampling strategy, response rate, and data analysis. Two reviewers independently conducted the quality assessments, and discrepancies in scoring were discussed and resolved collaboratively. In cases where consensus could not be reached, a third reviewer was consulted.

### 2.5. Ethics statement

Ethical approval was not required for this study since it relied solely on published articles and did not involve any human or animal intervention.

## 3. Results

The study selection process was rigorous and methodical. A search using Boolean operators, “(HPV OR human papillomavirus) AND (parent OR parent-child relations OR parent or child-parent relations) AND (vaccine OR papillomavirus vaccine OR wart virus vaccine) AND (Bahrain OR Kuwait OR Oman OR Qatar OR Saudi Arabia OR UAE OR United Arab Emirates)”, yielded 17 studies. After an initial review, 9 studies were excluded for various reasons: 4 did not include parents as the target group, and another 4 were conducted outside the GCC countries. One study conducted in Oman was not readily accessible for comprehensive analysis. Systematic reviews were also excluded to focus on original research. Consequently, 7 studies met all criteria and were included in the systematic review. No study was found in Oman, Bahrain, and Kuwait.

In the quality assessment, positive responses (“Y”) indicated that the respective criteria were met, while negative responses (“N”) denoted noncompliance. The evaluation of bias levels across the studies revealed varying degrees of susceptibility to bias. Specifically, Saqer et al demonstrated the lowest risk of bias with a score of 17. Studies by Alaamri et al, Alhusayn et al, Alshehri et al, and Hendaus et al exhibited a moderate risk of bias, each receiving a score of 16. In contrast, the studies conducted by Alkalash et al, Mohammad et al, and Tobaiqy et al showed the highest susceptibility to bias, with each receiving a score of 15.

**Figure 1.**
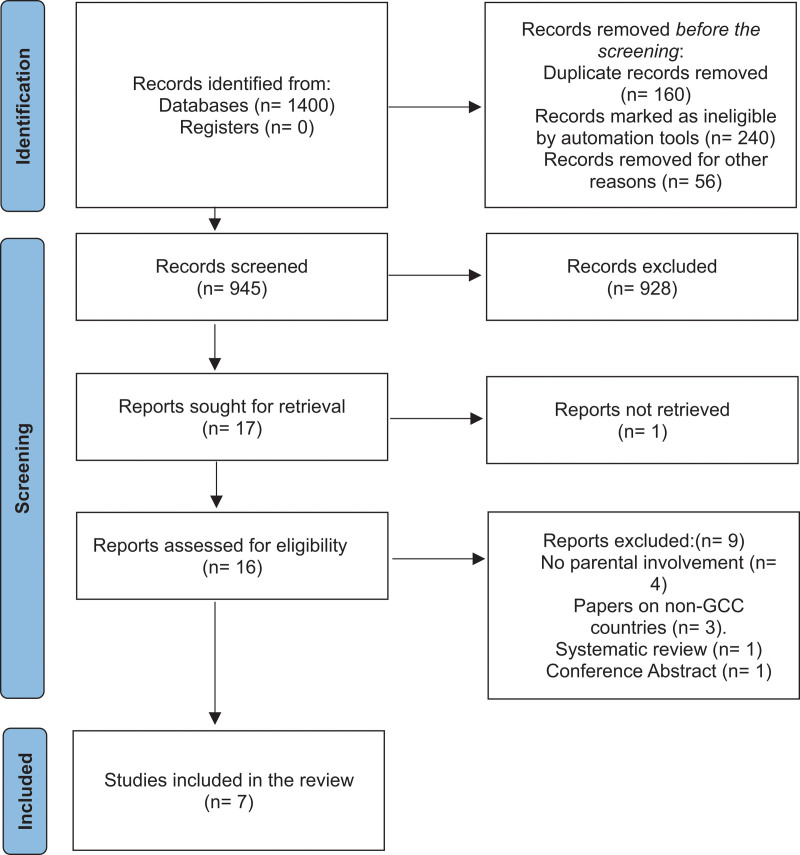
PRISMA flow chart.

The detailed PRISMA flow chart visually represents the study selection process (see Fig. 1). Initially, 1400 records were identified from databases. After removing duplicates and ineligible records, 946 records were screened. Out of these, 929 were excluded based on relevance. Seventeen reports were sought for retrieval, but 1 could not be accessed. The remaining 16 reports were assessed for eligibility, with 8 excluded for not meeting the criteria (e.g., not involving parents, conducted outside GCC, systematic reviews). Ultimately, 7 studies were included in the final systematic review, providing a robust foundation for analyzing parental perspectives on HPV vaccination in GCC countries (Fig. 1).

The systematic review identified 7 studies focused on parental awareness, knowledge, attitudes, and behaviors regarding HPV vaccination across Saudi Arabia, UAE, and Qatar. These studies varied in publication dates from 2017 to 2023, with a notable concentration in 2021, 2022, and 2023.^[5,12–16]^ The gender breakdown of participants across these studies revealed a consistent trend: mothers were significantly more represented than fathers.

Table 2 highlights the methodological variability among HPV vaccine studies in GCC countries, indicating diverse approaches in study designs, data collection methods, sample sizes, and study durations. All the studies included were cross-sectional studies. Additionally, Table 2 presents a summary of the 7 studies reviewed across the GCC countries, focusing on key findings related to knowledge and awareness about HPV and the HPV vaccine. The findings across these studies indicate a generally low level of awareness and knowledge about HPV and the HPV vaccine among participants in the GCC countries. The studies consistently show that a significant proportion of the population remains uninformed about the link between HPV and cervical cancer, as well as the availability and importance of the HPV vaccine. For instance, the study by Alhusayn et al^[12]^ reported that 70.6% of their respondents were unaware of the HPV vaccine. Similarly, Tobaiqy et al^[13]^ found that only 11% of their participants were aware of the link between HPV and cervical cancer, and a striking 96.8% were unaware of the HPV vaccine. On the other hand, the study by Alaamri et al^[16]^ showed a relatively higher awareness rate of 68.0% regarding HPV.

**Table 2:** Overview of the 7 studies included in the review across the GCC countries and key findings on knowledge/awareness.

Study	Design	Data collection	Sample size, response rate	Key findings	Duration
Alhusayn et al^[12]^	Cross-sectional survey	Interview-based	296, 77%	70.6% unaware of HPV vaccine.	November 2019–May 2020
Alshehri et al^[11]^	Cross-sectional survey	Self-administered	773, 66%	46.1% had poor knowledge of HPV.	December 2022–March 2023
Tobaiqy et al^[13]^	Cross-sectional survey	Online questionnaire	500, 78%	11% aware of HPV-cervical cancer link; 96.8% unaware of HPV vaccine.	June–August 2021
Saqer et al^[14]^	Cross-sectional survey	Self-directed	400, 77%	41.3% heard of HPV, 36.5% of vaccine.	February–April 2015
Alkalash et al^[15]^	Cross-sectional survey	Online questionnaire	343, 59%	Poor knowledge of HPV; 32.9% aware of vaccine.	June–November 2022
Alaamri et al^[16]^	Cross-sectional survey	Electronic	947, 70%	68.0% are aware of HPV.	September 2022–June 2023
Hendaus et al^[5]^	Cross-sectional survey	Self-administered	330, 70%	60.0% unaware of HPV-cancer link.	April 2019–March 2020

Table 3 presents a summary of the studies on parental attitudes and vaccination behaviors regarding HPV across different GCC countries, highlighting the prevailing attitudes and conclusions drawn from each study. The data in Table 3 indicates a generally poor attitude towards HPV vaccination among parents in Saudi Arabia, with high percentages of unwillingness or uncertainty about vaccinating their children. Studies such as those by Alhusayn et al^[12]^ and Tobaiqy et al^[13]^ highlight a significant reluctance among Saudi parents to vaccinate their daughters, with 89.5% and 94%, respectively indicating they did not receive or were unwilling to receive the HPV vaccine.

**Table 3:** Parental attitudes and vaccination behaviors in HPV studies.

Study	Country	Attitude towards parent HPV vaccination	Conclusion
Alhusayn et al^[12]^	Saudi Arabia	89.5% did not receive the HPV vaccine for themselves or their children.	Poor attitude
Alshehri et al^[11]^	Saudi Arabia	44.5% of parents had the intention to vaccinate their daughters. 43.6% were not sure about vaccinating their daughters. 11.9% did not intend to vaccinate their daughters.	Poor attitude
Tobaiqy et al^[13]^	Saudi Arabia	94% were unwilling to vaccinate their daughters.	Poor attitude
Saqer et al^[14]^	UAE	76.6% of parents were willing to vaccinate their daughters. The percentage of willingness increased to 92.9% if the Ministry of Health (MOH) recommended the vaccine.	Good attitude
Alkalash et al^[15]^	Saudi Arabia	Only 7.2% had vaccinated their female children.	Poor attitude
Alaamri et al^[16]^	Saudi Arabia	Saudi acceptability of the vaccine was 0.671 times lower than that of non-Saudis.	Poor attitude
Hendaus et al^[5]^	Qatar	About 20% of parents were not convinced about the HPV vaccine.	Good attitude

In contrast, findings from the UAE (Saqer et al) and Qatar (Hendaus et al) present a more favorable attitude towards HPV vaccination. In the UAE, 76.6% of parents expressed willingness to vaccinate their daughters, a figure that increased to 92.9% with official health recommendations. Similarly, in Qatar, approximately 20% of parents showed reluctance, indicating that a majority were either convinced or neutral about the vaccine.

Table 4 outlines the major barriers and concerns contributing to hesitancy towards HPV vaccination across various GCC countries based on the studies reviewed. The primary barrier identified in the study by Alhusayn et al^[12]^ in Saudi Arabia was a lack of awareness about the HPV vaccine. Alshehri et al^[11]^ also found in Saudi Arabia that concerns about the effectiveness and safety of the vaccine, coupled with a general lack of knowledge about HPV, were significant deterrents. Tobaiqy et al^[13]^ reported that the main reason for vaccine refusal was insufficient information on the importance of HPV vaccination. Similarly, Alkalash et al^[15]^ highlighted that a frequent barrier was individuals’ confidence in not being at risk of contracting HPV.

**Table 4:** Barriers towards HPV vaccination.

Study	Country	Major barriers/concerns/reason for hesitancy
Alhusayn et al^[12]^	Saudi Arabia	Not aware of the vaccine.
Alshehri et al^[11]^	Saudi Arabia	Worries about the effectiveness and safety of the HPV vaccine as well as lack of knowledge about HPV.
Tobaiqy et al^[13]^	Saudi Arabia	The main reported reason behind refusing the vaccine was the dearth of information on the benefits of HPV vaccination.
Saqer et al^[14]^	UAE	Individuals who declined vaccination cited reasons such as believing they were too old or having concerns about the vaccine’s safety.
Alkalash et al^[15]^	Saudi Arabia	The most common barrier for vaccination was their confidence of being not at risk.
Alaamri et al^[16]^	Saudi Arabia	Doubted the safety of the vaccine.
Hendaus et al^[5]^	Qatar	They doubted the safety of the vaccine and believed it was developed primarily for marketing purposes.

In the UAE, Saqer et al^[14]^ found that those who refused vaccination often cited age, believing they were too old, and had safety concerns about the vaccine. In Qatar, Hendaus et al^[5]^ noted that doubts about the vaccine’s safety and suspicions that it was developed primarily for marketing purposes were prevalent. Alaamri et al further supported these findings in Saudi Arabia, identifying safety concerns as a major barrier.

## 4. Discussion

This systematic review examined existing research on parental knowledge, attitudes, and barriers to HPV vaccination in the GCC region. According to our review findings, the level of awareness in the 7 studies was found to be low to high. These studies collectively illustrate a range of awareness levels, from as low as 11% awareness of the HPV-cervical cancer link^[13]^ to as high as 68% general awareness of HPV.^[16,17]^ Most studies, however, highlighted a significant lack of knowledge about HPV and its vaccine, indicating a need for enhanced educational efforts in the region. Additionally, mothers generally have higher knowledge than fathers.^[12,13,16]^ The findings of these studies align with research conducted in China, which revealed that the majority of mothers had higher awareness about HPV.^[18]^ Similarly, a systematic analysis revealed a range of awareness levels from poor to high among mothers in African countries.^[19]^ The higher prevalence of knowledge about HPV among mothers compared to fathers may be attributed to the differential involvement in caregiving responsibilities. Mothers tend to be more actively engaged in childcare activities and healthcare decision-making.

In 2018, Gamaoun performed a comprehensive analysis that encompassed 18 studies undertaken in nine Arab nations.^[20]^ The level of knowledge and awareness regarding HPV infection varied from 4.2% to 97.0% across different states and subpopulations. The highest percentage was observed in the subgroup consisting of clinicians (97.0%), while the lowest percentages were found in the grouping of parents (ranging from 4.2–18.0%). Parents had a poor level of knowledge and awareness, as low as 8.4%, regarding the causal relationship between HPV infection and cervical cancer. The proportion of HPV vaccine awareness among parents varied between 14.2% and 34.2%, depending on the country. Nearly 99% of parents expressed their acceptance of the immunization. Similar to the findings of this systematic review, the variations in knowledge and awareness levels highlight the critical need for targeted educational interventions to improve HPV vaccination uptake.

Additionally, according to our review findings, parental concerns regarding HPV vaccination included perceived pain, lack of medical recommendation, doubts about vaccine effectiveness and safety, perceptions of daughters being too young or old, cost concerns, and Skepticism about the vaccine’s purpose or marketing strategy, considering it an old cancerous disease contributing to hesitancy toward HPV vaccination.^[5,12–14,21]^ Vaccination hesitancy in other countries such as China, the United States of America, and Europe included the cost of vaccination, poor knowledge, lack of medical recommendations, and concerns about vaccine safety.^[22–25]^ The variations in parental concerns regarding HPV vaccination between our review findings and other countries such as China, the United States, and Europe may arise from differences in cultural perceptions, healthcare systems, and regional factors prompting attitudes towards vaccine safety, cost considerations, and medical recommendations.

Culture and religion also influence parents’ HPV vaccine decisions.^[26]^ According to our review, in UAE and Qatar studies, women who believed the HPV vaccine was unnecessary for their children often based their decision on religious and cultural beliefs.^[5,21]^ A study in Thailand, with a religious population, also found that parents’ knowledge and beliefs were associated with accepting HPV vaccination for their daughters based on their cultural and religious beliefs.^[11]^ In Islamic countries, people follow Islamic teachings and according to Islam, they cannot have sex before marriage.^[27]^ Therefore, these people consider that vaccine is unnecessary before marriage because they are not going to have sex before marriage. This perception of the vaccine is further amplified by concerns about discussing sexual health with minors, which directly contributes to low vaccination rates across the region.^[28,29]^ Parents do not like to talk about sex in front of minors. Similar to these beliefs, participants in our studies likely believed that their daughters were less likely to get cervical cancer.

In Islamic countries, another significant concern pertains to the necessity of immunizations that the products should be verified as halal.^[30]^ According to Islamic jurisprudence in Kuwait, Qatar, Saudi Arabia, UAE, Oman, and Bahrain, Muslims must abstain from using any medicinal substances or ingredients derived from haram sources, which include those derived from pigs or their derivatives.^[31]^ Therefore, most Muslims do not get vaccinated because they believe that the vaccine is obtained from experimentation with pigs. When there is uncertainty over the “halal” certification of a vaccine, parents frequently resort to alternative or homeopathic remedies.^[31]^ This propensity may be confirmed in the research conducted by Zuzak et al since their study revealed a correlation between the use of complementary medicine and decreased vaccination rates.^[32]^ Overall, the beliefs about premarital sex and using halal products leads to decreased vaccination rates in Muslim countries.

Although, several GCC nations have introduced the HPV vaccine into their national immunization schedules, highlighting its role in preventing HPV-related illnesses, notably cervical cancer.^[33]^ However, adoption varies across the region, with some countries achieving high vaccination coverage while others are still in the early stages.^[33]^ Cultural beliefs, misconceptions, and logistical challenges, especially in remote areas, pose obstacles.^[33]^ Rural areas often have lower coverage compared to urban regions, indicating an uneven distribution of the vaccine.^[33]^

The GCC recognizes the imperative of HPV vaccination, aiming for consistent and comprehensive protection across member states.^[33]^ To achieve this, implementing cohesive regional policies for equal vaccine access is crucial, involving the establishment of distribution protocols, financial support, and robust monitoring systems to effectively track immunization rates.^[33]^ Web-based initiatives, such as vaccine websites with interactive social media features, can enhance pediatric and maternal vaccine acceptance.^[34]^ Collaborations among governmental entities, healthcare providers, and international organizations can contribute to a more integrated approach to implementing HPV vaccination initiatives.^[34]^ Customized outreach initiatives, like mobile vaccination units or vaccines in local pharmacies, can improve accessibility in rural GCC regions.^[35]^ Additionally, there is a need to address religious concerns, social norms, and misconceptions about HPV and its vaccine in each GCC country.^[35]^ To encourage this, multiple formats like brochures, videos, and social media campaigns can be used to reach diverse audiences.^[35]^ Similarly, trusted figures like imams, community elders, and nurses need to be informed about the necessity of HPV vaccination to disseminate accurate information and address vaccine hesitancy within their communities.^[35]^ Training of healthcare providers should be done on effective communication strategies to address individual concerns and build trust with patients.^[35]^

Additionally, the integration of HPV vaccination education into school curricula with age-appropriate materials and interactive activities can also boost vaccine uptake.^[1,35]^ Partnerships with teachers and school administrators need to create a supportive environment for vaccination.^[35]^ GCC governments are expected to intensify efforts, directing resources towards awareness campaigns and possibly making the HPV vaccine mandatory for adolescents.^[2,34]^ Collaborations with global health organizations and vaccine manufacturers are poised to significantly impact knowledge dissemination and vaccine accessibility.^[5,34]^ Effective coordination, involving establishing unified vaccination strategies, sharing best practices, and standardizing policies, is crucial for success.^[34]^ Encouraging data and expertise exchange among member states and global health bodies can optimize resource allocation and streamline vaccination efforts.^[11,34]^ Leveraging technological advances, such as mobile health applications, and private sector participation can enhance the distribution of vaccines, especially in inaccessible regions.^[15]^

In conclusion, this systematic review explores parental knowledge, attitudes, and barriers to HPV vaccination in GCC countries. It shows differences in HPV-cervical cancer knowledge and vaccination, with some studies showing both positive and negative attitudes and others showing better acceptance rates due to vaccine safety and efficacy. The identified barriers to HPV vaccination include safety concerns, efficacy doubts, age-related hesitancy, and insufficient knowledge. Tailored interventions addressing specific barriers within GCC are crucial, but limitations, including regional predominance and methodological variations, necessitate standardized protocols for future research to enhance the comparability and reliability of outcomes. Despite moderate HPV awareness and positive attitudes in some GCC countries, substantial knowledge gaps and vaccine hesitancy persist. To bridge this gap, targeted public education, effective healthcare communication, and collaboration with community leaders are important. By addressing misinformation and developing trust, GCC can pave the way for increased HPV vaccination uptake and protect the health of generations to come. Additionally, the scarcity of data from some GCC countries emphasizes the need for more comprehensive studies.

## Author contributions

**Conceptualization:** Najim Z. Alshahrani.

**Data curation:** Najim Z. Alshahrani, Jaber Abdullah Alshahrani, Fahad Marzooq Alshammri, Wael Saeed Alshahrani, Ahmed Ayed Hadi Alzabali, Abdulrahman Ahmed Alshehri, Nasser Z. Alduaydi.

**Formal analysis:** Najim Z. Alshahrani, Badur Saad Almushari, Wael Saeed Alshahrani, Abdulrahman Ahmed Alshehri, Khalid Alotaibi.

**Funding acquisition:** Najim Z. Alshahrani, Jaber Abdullah Alshahrani, Badur Saad Almushari, Fahad Marzooq Alshammri, Wael Saeed Alshahrani, Ahmed Ayed Hadi Alzabali, Abdulrahman Ahmed Alshehri, Nasser Z. Alduaydi, Manea Alqarni, Ammar Mohammed A. Alamri.

**Investigation:** Najim Z. Alshahrani, Jaber Abdullah Alshahrani, Badur Saad Almushari, Fahad Marzooq Alshammri, Wael Saeed Alshahrani, Ahmed Ayed Hadi Alzabali, Abdulrahman Ahmed Alshehri, Nasser Z. Alduaydi, Manea Alqarni, Ammar Mohammed A. Alamri, Khalid Alotaibi.

**Methodology:** Najim Z. Alshahrani, Jaber Abdullah Alshahrani, Badur Saad Almushari, Manea Alqarni.

**Project administration:** Najim Z. Alshahrani, Wael Saeed Alshahrani.

**Resources:** Najim Z. Alshahrani, Jaber Abdullah Alshahrani, Ahmed Ayed Hadi Alzabali, Ammar Mohammed A. Alamri, Khalid Alotaibi.

**Software:** Najim Z. Alshahrani, Jaber Abdullah Alshahrani, Badur Saad Almushari, Khalid Alotaibi.

**Supervision:** Najim Z. Alshahrani, Badur Saad Almushari.

**Validation:** Najim Z. Alshahrani, Fahad Marzooq Alshammri, Ahmed Ayed Hadi Alzabali, Abdulrahman Ahmed Alshehri, Nasser Z. Alduaydi, Manea Alqarni.

**Visualization:** Najim Z. Alshahrani, Khalid Alotaibi.

**Writing – original draft:** Najim Z. Alshahrani, Jaber Abdullah Alshahrani, Badur Saad Almushari, Fahad Marzooq Alshammri, Wael Saeed Alshahrani, Ahmed Ayed Hadi Alzabali, Abdulrahman Ahmed Alshehri, Nasser Z. Alduaydi, Manea Alqarni, Ammar Mohammed A. Alamri.

**Writing – review & editing:** Najim Z. Alshahrani, Jaber Abdullah Alshahrani, Badur Saad Almushari, Wael Saeed Alshahrani, Nasser Z. Alduaydi, Manea Alqarni, Khalid Alotaibi.
